# Heat Perceptions, Behaviors, and Barriers of Unhoused Individuals in Germany

**DOI:** 10.1002/puh2.70408

**Published:** 2026-10-06

**Authors:** Philipp Sprengholz, Robert W. Bruckmann, Linnea Noomi Daniel, Elisabeth Luna Else, Lilliana Babette Moreth, Nicole Piatti

**Affiliations:** ^1^ Institute of Psychology University of Bamberg Bamberg Germany; ^2^ Institute for Planetary Health Behavior University of Erfurt Erfurt Germany; ^3^ Health Communication Working Group Bernhard Nocht Institute for Tropical Medicine Hamburg Germany

**Keywords:** extreme heat, heat–health action plans, heat‐protective behavior, homelessness

## Abstract

Heat waves pose critical health risks for unhoused people. However, little is known about how this population perceives and copes with heat, particularly in Europe. Employing a sequential mixed‐methods approach, we investigated dimensions of unhoused individuals’ heat perceptions, coping behaviors, and barriers via semi‐structured interviews (*N* = 9) to inform the design of a quantitative survey administered across 13 German cities in 2024 (*N* = 252). Results revealed that many homeless people experience fatigue, nausea, or faintness on hot days, whereas only half of respondents considered heat‐related health problems likely or dangerous. Protection was most strongly associated with perceiving that people important to participants protect themselves. At the same time, protective behavior was structurally constrained by limited opportunity, including inadequate access to hydration and cooling spaces, eviction, and lack of cool and secure places to store medication. Overall, the results warrant urgent policy interventions, such as improving access to hydration resources, establishing dedicated cooling shelters, and suspending evictions from cool places in the case of official heat warnings. Furthermore, information‐based interventions should raise awareness regarding heat‐related health risks and protective actions among unhoused populations, stakeholders working with them, and the general public.

## Graphical abstract

Across in‐depth interviews and a survey of 252 unhoused people in 13 German cities, many underestimated heat risks yet often experienced heat‐related fatigue, nausea, or faintness. Participants reported limited hydration and frequent eviction from cooling spots, heat‐aggravating medication whose dosage and storage were rarely adapted, and minimal sun protection. The findings point to dedicated cooling shelters, better hydration and toilet access, and targeted heat‐risk awareness.

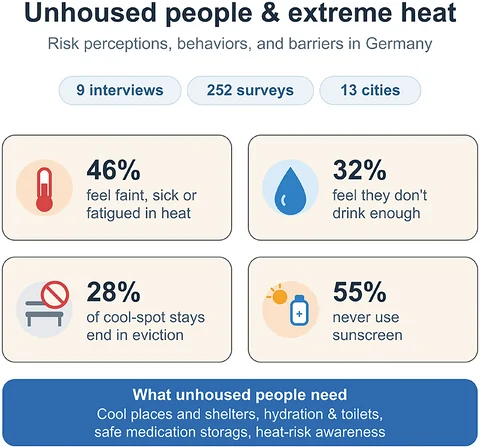

## 1 Introduction

Heat waves are becoming more frequent and intense across the globe [1, 2]. In Germany, the number of days per year with temperatures exceeding 30°C has multiplied in recent decades [3]. Heat waves contribute to morbidity and mortality through various mechanisms, for instance, by increasing dehydration [4], causing heat‐related illnesses (such as heat exhaustion, hyperthermia, or heat stroke; [5]), worsening cardiovascular and respiratory conditions [6], or provoking renal failure [7]. As a consequence, an estimated 62,775 people died from heat in Europe during the summer of 2024, the warmest on record [8].

Previous research shows that heat waves pose greater risks for those with preexisting medical conditions [9, 10], significant exposure to heat (e.g., outdoor workers; [11]), or aging ([12]; though evidence also points toward a growing risk for younger populations, see [13]). Multiple risk factors converge for unhoused individuals [14], here understood as people living in rooflessness or houselessness—the first two of the four conceptual categories of the European Typology of Homelessness and Housing Exclusion (ETHOS; [15]), covering those living in public spaces, in night shelters, or in temporary or supported accommodation for homeless people. This population suffers from chronic health issues above average [16], lacks stable shelter, and has limited access to fundamental resources, such as water or adequate clothing [17, 18]. Direct evidence on the resulting mortality is just emerging: Analyzing deaths between 2015 and 2022, Lin et al. [19] attributed 50% of deaths among unhoused people in Clark County, Nevada, and 7% in Los Angeles County, California, to daily temperature, mostly to heat rather than cold, with effect estimates orders of magnitude larger than those observed for the general population.

Although housing provision is often considered the most important strategy for reducing unhoused people's vulnerability [18], short‐term strategies must resort to simpler, more affordable measures, such as targeted health information, water provision, or dedicated cooling spaces, to reduce the risks associated with extreme heat. To identify and effectively implement promising interventions, objective needs and subjective perceptions shaping behavior‐bound heat vulnerability need to be understood in depth. In line with prominent psychological theories, such as the Theory of Planned Behavior [20], the Health Action Process Approach [21], or the Protection Motivation Theory [22], the intention to adopt health‐promoting behaviors depends on factors, like the individuals’ knowledge and outcome expectancies, social norms, beliefs, attitudes, external cues to action, and peer behavior. However, intention alone does not sufficiently predict action [23]. The Capability, Opportunity, Motivation‐Behavior (COM‐B) model [24] places these motivational factors alongside two further conditions: Individuals must be capable of performing a behavior and have the opportunity to do so. Building on that, engaging in heat protection requires knowing of the individual dangers associated with extreme heat and how to adopt preventive behaviors (psychological capability), have actual access to mitigation resources (physical opportunity), and be supported by social norms and peer behavior (social opportunity). Motivation may stem from reflective processes, such as perceiving a threat and one's own capability to act, as well as from the automatic pathway via habits and emotional responses.

There has been some research on heat perceptions and adaptive behaviors of unhoused populations in the United States and Australia [18, 25, 26]. For example, Robertson et al. [27] surveyed unhoused and housed residents of Columbia, South Carolina, and found that unhoused respondents rated the severity of heat impacts higher than housed respondents, with subjective norms, perceived behavioral control, the perceived value of heat information, and difficulty comprehending that information predicting the intention to avoid heat. Cronley et al. [28], interviewing 42 people experiencing homelessness in a mid‐sized city in the southeastern United States, showed how social exclusion in the built environment erodes adaptive capacity by limiting scarce resources and restricting mobility. Thompson et al. [29] measured overnight temperatures inside tents and found that trapped heat left them up to 5°C warmer than ambient air, with tent siting for shade and water constrained by policing and displacement. Together, these findings indicate that heat protection among unhoused people is limited less by awareness of the danger than by whether the surrounding environment permits protective action. European perspectives, however, are largely missing in the literature. Given the differing climates, political contexts, and welfare systems, we aimed to investigate the experiences of unhoused people in Germany. In two sequential studies, we explored the heat perceptions and adaptive behaviors of this group and investigated behavioral barriers to impactful heat mitigation along with derived strategies to strengthen resilience. Study 1 carved out relevant topics and questions using in‐depth semi‐structured interviews, informing the design of Study 2, a survey quantifying perceptions, actions, and problems in a larger unhoused population across Germany.

## 2 Study 1: Qualitative Exploration

Study 1 entailed semi‐structured interviews to gain insights into unhoused people's perceptions, adaptive actions, and major barriers that prevent protective behavior.

### 2.1 Methods

#### 2.1.1 Participants

Participants were recruited from medical facilities delivering services to unhoused individuals in Hamburg and Nuremberg between March and April 2024. In total, nine individuals completed the interviews (F82, F68, M45, and M30 in Nuremberg, and F45, M65, M57, M54, and M40 in Hamburg). Participants were between 30 and 82 years old, and the cohort comprised six male and three female participants. (Gender and age are encoded in the participant identifier.) All participants had already been unhoused the previous summer. All interviews were conducted in German, each interview took about 30 min, and the participants received €10 in cash as compensation.

#### 2.1.2 Procedure

Participants were interviewed separately in a quiet, private setting. Because of the participants’ anonymity concerns, we decided against taping the interviews and instead recorded their answers on paper following Rutakumwa et al. [30]. Right after the interviews, the notes were systematically structured and summarized.

The interview guide included various open questions about various aspects of participants’ experiences, such as how they spent hot days and nights (e.g., *What did a typical hot day in summer look like for you?*), how they protected from sun and heat (e.g., *How do you typically get water?*), what sources of information they used or needed (e.g., *How do you find out that it's going to be hot the next day?*), what barriers prevented protective behavior (e.g., *Can you protect yourself from direct sun and heat if you want to? What makes it difficult for you?*), how other people treated them (e.g., *How do other people behave towards you on hot days, for example, passerby or authorities?*), and how the heat affected preexisting medical conditions (e.g., *Do you have any pre‐existing conditions that you notice more in hot weather?*). We also asked about positive past experiences or support systems (e.g., *Were there things or people particularly supporting you, making the heat bearable for you?*) and wishes for the future regarding potential improvements or heat mitigation strategies (e.g., *What changes would you like to see in the city?*). The entire original interview guide is available in the https://doi.org/10.17605/OSF.IO/RKWAM.

#### 2.1.3 Analyses

The structured notes were analyzed using structuring qualitative content analysis with deductive (concept‐driven) category formation [31]. Coding categories were defined a priori from the domains of the interview guide. Statements from the notes were assigned to these categories in joint sessions, with any divergence resolved by discussion until consensus was reached. No additional categories were derived inductively. Medications named by participants were classified as heat‐relevant on the basis of published evidence that they increase core temperature responses during heat stress (e.g., ACE inhibitors and diuretics; [10]) or are subject to heat‐altered pharmacokinetics [32]. Because coding was collaborative rather than independent, and the notes were structured summaries rather than verbatim transcripts, no measure of intercoder agreement was computed.

### 2.2 Results

#### 2.2.1 Heat Experience, Disease, and Medication

Participants experienced considerable discomfort due to summer heat (F82, F68, F45, and M40) and particularly complained about extreme temperature fluctuations between day and night (F45). Participants reported various infections and health conditions, such as cardiovascular diseases and hypothyroidism, with worsening symptoms on particularly hot days, such as increasing breathing problems amidst heat waves (F82). Three participants (F82, F68, and M57) reported taking medications that are known to increase heat‐associated health risks by reducing thirst (e.g., ACE inhibitors) or dehydrating the body (e.g., diuretics). Despite the impact of heat on pharmacokinetics, none of the participants reported adapting their medication dosages during hot days. Furthermore, participants often carried their drugs in person instead of storing them in cool places as instructed on many package inserts (F82, F68, and M57). Varying reasons for such practices included the requirement of frequent medication intake (F68), absence of adequate storage options (M57), or participants’ fear regarding the theft of their drugs when leaving them unattended (F82).

#### 2.2.2 Protective Behaviors and Barriers

##### 2.2.2.1 Clothing

Some participants wore caps and sunglasses to protect themselves from sun exposure and heat (F82 and M45), whereas others did explicitly not (M57 and M30). To adapt to fluctuating temperatures, some participants carried extra clothes in bags and backpacks (M65, M57, and M54), whereas others changed clothes in clothing banks (F45). Two participants (M57 and M30) stored their clothes in a garage or self‐storage facility outside the city, necessitating long‐distance commutes to change clothes between hot days and cold nights. One participant (M57) indicated spending hot days shirtless due to the lack of access to alternative clothing options. Exposure to the sun was considered unproblematic by several participants (M65, M57, and M30).

##### 2.2.2.2 Hydration

Participants employed different strategies to get drinking water. Some used free offers from charitable organizations, such as soup kitchens or railway missions (F82, F68, F45, and M54), whereas others refilled bottles in public drinking fountains, shopping centers, and public restrooms (M65 and M30). Some participants reported the need to purchase bottled water, especially on weekends when many charities were closed (F45 and M40). Even though all participants recognized the importance of hydration on hot days, some had deliberately adopted limited water intake so that they could avoid using rare or expensive public toilets (F68, M57, and M45). For instance, one participant explained that public toilets were far from places where she used to beg for money. Additionally, reaching the toilet was difficult due to the need to transport many of her belongings and the lack of shade or shelter alongside the route (F68).

##### 2.2.2.3 Daytime Cooling Strategies

Water was not only used for drinking but also for cooling. Multiple participants reported refreshing themselves by wetting their face (M57) or moistening a cloth to place on the neck (F68 and M30). On extremely hot days, participants sought out various cool places, such as railway stations (F82 and F45), churches (M45), shopping malls and supermarkets (M65 and M45), libraries (M30), charitable organizations (F82, M40, M45, and M30), and shaded spots in public parks and pedestrian zones (F82, F68, M45, and M30). Sometimes, air‐conditioned public transport services were used to avoid the heat (F82 and F68). Some participants resorted to streams and swimming lakes to cool down (M57 and M45), whereas one woman reported regular traveling to Kneipp water‐treading basins in surrounding villages, which were free to use and empty during workdays (F68). Participants complained about the scarcity of safe, cool places to sit and lie down, especially on weekends when many charities, businesses, and public buildings are closed (F68, F45, M45, and M30). But even when places were open, participants often did not feel welcome (M57) or were forcibly evicted by residents, shop owners, police, and regulatory authorities (F68, F45, M57, M54, M45, and M30). One participant (M30) also faced a social dilemma: He had found good places to spend hot days and wanted to share them with other unhoused people but refrained from doing so because he feared that eviction from the place was more likely in the presence of a higher number of unhoused people.

##### 2.2.2.4 Overnight Stays

Participants spent summer nights in various locations, including railway stations (M54), storage facilities (M65), and bank branches (M30). Some slept outdoors (M54 and M40) or in a car (M57). Only one woman regularly stayed in an overnight shelter (F82). Some participants complained that shelters were closed in the summer months (F45) or that high indoor temperatures prevented a good sleep in the shelters (F82 and M45). One man (M45) acknowledged greater comfort in sleeping outside but expressed fear about the risk of being robbed.

#### 2.2.3 Information Use and Needs

Participants predominantly learned about upcoming heat waves from public weather displays in pedestrian areas, railway stations, and public transport vehicles (F82, M65, and M30). Some used weather apps on their mobile phones (F68, M65, and M45), and others were informed by charity employees, for example, people working in railway missions (F45). Although participants felt well‐informed about the weather status, they were missing guidance regarding how to cope with the extreme heat. For instance, two men (M45 and M30) expressed the need for additional up‐to‐date information on locations with easily accessible cooling areas and free drinking water.

### 2.3 Discussion

The interviews revealed different mechanisms through which unhoused people suffer from and struggle with summer heat. High temperatures exacerbate preexisting health problems, and necessary medication dosage and storage adjustments are rarely made. Access to cool places is rare, and citizens and law enforcement often evict unhoused people. Staying hydrated poses a daily challenge as free water supplies are lacking or unknown, and some unhoused people prefer to limit their water intake to reduce toilet visits as far as possible. In some cases, inadequate clothing also becomes a challenge, prompting the risk of hyperthermia (if clothing is too thick) or sunburn (if the skin is not covered adequately). To summarize, the in‐depth interviews clarified different pathways and details on how extreme heat can intensify the vulnerability of unhoused people.

The discussion of Study 2 details how our findings relate to and extend existing literature. For now, several limitations should be considered. The interviews took place in spring, so accounts of heat experiences were retrospective and may have been less salient than they would have been during or shortly after a heat wave. The sample was small and recruited through medical facilities in two cities, so people not in contact with such services are not represented. As participants objected to audio recording, interviews were documented in written notes rather than verbatim transcripts, which precluded verbatim quotation and a formal measure of intercoder agreement. The wide range of topics covered within approximately 30 min limited the depth attainable on any single theme. Study 2 addresses several of these constraints by surveying a larger and geographically broader sample during a summer season.

## 3 Study 2: Quantitative Survey

On the basis of the findings of Study 1, we conducted a paper–pencil survey to quantify and interrelate heat‐related perceptions, coping behaviors, and problems faced by unhoused people. We further investigated sun protection attitudes and behaviors, as the interviews indicated a strong connection between heat and sun protection.

### 3.1 Method

#### 3.1.1 Participants

Between July and September 2024, we visited 15 day centers for unhoused people in 13 German cities (Berlin, Dortmund, Erlangen, Frankfurt am Main, Freiburg, Halle, Hannover, Hamburg, Leipzig, Ludwigsburg, München, Nürnberg, and Zwickau), spending 1 day for each center. Unhoused individuals present at the centers were invited to participate in the survey and received an incentive worth €5 in cash upon completion of the questionnaire. In total, *N* = 252 individuals participated in this study. The study participants were 18–84 years old (*M* = 46.98, SD = 13.45). The majority (76%) reported being male; 23% indicated female gender. The year in which participants were unhoused for the first time ranged between 1986 and 2024, and 69% reported having health insurance.

#### 3.1.2 Measures

The questionnaire was developed with support and input from various experts working for homeless shelters and the National Federation for the Homeless in Germany. As no validated instrument covering heat perceptions, coping behaviors, and barriers was available that fits the survey‐related procedural requirements in this population, all items were newly developed for this study. Their content was derived from the themes identified in Study 1, ensuring that the questionnaire covered the experiences, behaviors, and barriers that participants themselves had raised. Items were formulated in simple language, and short answer scales were used whenever possible to facilitate the participation of people with reading difficulties. Most constructs were assessed with single items or two‐item measures in order to keep the questionnaire short and cognitively accessible. The original German version of the questionnaire is provided in the https://doi.org/10.17605/OSF.IO/RKWAM. If necessary, researchers read the questions aloud and helped participants fill in their answers. The questionnaires were digitized by two authors, and uncertainties were discussed and resolved with the involvement of a third author.

##### 3.1.2.1 Risk Perceptions

Participants were asked about the probability and severity of heat‐related health problems by rating two statements (*Heat probably leads to health problems for me*, and *Health problems caused by heat are dangerous for me*) on a 4‐point agreement scale (ranging from *strongly disagree* to *strongly agree*). They were also asked about the severity perceptions of people important to them (*People who are important to me consider heat dangerous*) using the same agreement scale.

##### 3.1.2.2 Heat Experience and Protection

Participants were asked if they experienced fatigue, nausea, or faintness because of heat since becoming unhoused, and their answers were recorded on a binary scale (*yes/no*). They were further asked how much they agreed with the statements “I protect myself from heat*”* and “People who are important to me protect themselves from heat*”* on a 4‐point scale ranging from *strongly disagree* to *strongly agree*.

##### 3.1.2.3 Medication

Participants were asked whether they regularly take medication. If they indicated affirmative responses, they were further asked to write down each medication's name or the condition it intended to help with. Answers were recoded into a binary variable indicating if the medication is known to increase heat‐related risks (e.g., by decreasing thirst or increasing dehydration), according to classifications by Haefeli and Czock [33]. Participants were further asked if they could adapt the dosage on hot days and if they had access to a cool place for storing medication if needed (*yes/no*). They could also indicate whether they carry medication with them because they have to take it regularly (*yes/no*) or out of concern that it might otherwise be stolen or lost (*yes/no*).

##### 3.1.2.4 Use of and Eviction From Cool Places

Participants were presented with a list of nine potential cooling places (train and metro stations, stores and shopping centers, public buildings, such as libraries and government offices, churches, shady spots along streets and in pedestrian zones, shady spots in public parks, public transport services, swimming pools, and bathing spots by rivers and lakes). For each location, they were asked if they used it to cool down during high temperatures (*yes/no*) and if police, employees, or local residents had ever evicted them on hot days (*yes/no*).

##### 3.1.2.5 Hydration

Participants were asked about their intake of non‐alcoholic beverages (such as water, juice, lemonade, coffee, or tea) consumed the day before. They could indicate the amount of beverages by marking one or multiple container pictograms representing different sizes (250 mL glasses, 500 mL bottles, 1 L cartons, and 1.5 L bottles). The total fluid intake (in mL) was summed up and stored as a new variable. Participants were further asked if they were drinking enough fluids (*yes/no*). If this was not the case, they could mark one or multiple reasons from the following options: *I can't afford any more drinks*, *There are no free drinks (e.g., drinking fountains, drinks dispensers) nearby*, *I don't like the taste of free drinks (e.g., drinking fountains, drinks dispensers)*, and *If I drink more, I will have to go to the toilet more often, which is too inconvenient or too expensive*.

##### 3.1.2.6 Clothing and Sun Protection

Participants were asked if they had enough clothes, if they could store unused or unwanted clothes (e.g., winter clothes), and if they could wash their clothes in the summer (*yes/no*). Participants’ sunburn frequency for this year was assessed through a categorical scale (never, one, two, three times, or more often). They were further asked how often they were wearing a hat, airy clothing, long sleeves, and sunscreen to protect from heat and sun using a 3‐point scale (*never, sometimes, often*). Participants’ agreement to the four sun‐related statements—*Sunburn is dangerous for me*, *Sunscreen helps me to protect myself from sunburn*, *I can afford sunscreen*, and *I find the use of sunscreen pleasant—*was assessed on a 4‐point scale with responses ranging from *strongly disagree* to *strongly agree*.

##### 3.1.2.7 Overnight Stay

Participants were asked to specify where they spent hot nights by choosing one or multiple places, including shelter accommodation, homes of friends or relatives, tents, cars, open‐air locations, or elsewhere. Sleeping quality was assessed on a 4‐point scale from *very bad* to *very good*.

#### 3.1.3 Analyses

Analyses were descriptive and exploratory. Frequencies, percentages, means, and standard deviations were computed for all items. The perceived severity of heat risks for participants and people important to them was compared using a paired‐samples *t*‐test with Cohen's *dz* as effect size, and the gender difference in restricting fluid intake to avoid toilet visits was tested using a chi‐square test of independence. Two multiple linear regressions predicted self‐reported heat protection from the perceived likelihood and severity of heat risks and the observed protective behavior of others important to participants and sleeping quality from the places where participants spent hot nights. Sunscreen use was modeled by ordinal logistic regression with perceived sunburn risk, sunscreen efficacy, affordability, and pleasantness of use as predictors, reported as odds ratios. Bivariate associations were quantified using correlations. All tests were two‐tailed with *α* = 0.05, each analysis used the cases available for the variables involved, and full model outputs are reported in the https://doi.org/10.17605/OSF.IO/RKWAM. Analyses were conducted in R (version 4.3.1). Medications named by participants were classified for heat relevance as in Study 1.

### 3.2 Results

#### 3.2.1 Risk Perceptions, Protection, and Dangerous Experiences

As shown in Figure 1, 56% of participants perceived heat‐related health risks as probable (*M* = 2.67, SD = 1.06), and 46% considered them severe (*M* = 2.43, SD = 1.08). Own severity ratings were lower compared to perceived severity considerations of others important to participants (*M* = 2.81, SD = 1.04, *t*(232) = −4.38, *p* < 0.001, *d* = −0.29). The majority of participants (76%) reported protecting themselves from heat (*M* = 3.12, SD = 0.95); a multiple linear regression on risk perceptions and others’ protective behavior (*R* ^2^ = 0.26) revealed that self‐reported protection was associated with perceiving that people important to them protect themselves from heat (*b* = 0.50, SE = 0.06, *p* < 0.001), and when the perceived heat‐related health risks were rated as more severe (*b* = 0.14, SE = 0.06, *p* = 0.018), but not when they were perceived as more likely (*b* = 0.09, SE = 0.06, *p* = 0.150; see https://doi.org/10.17605/OSF.IO/RKWAM for details). Although nearly half of the respondents (46%) reported having experienced heat‐related fatigue, nausea, or fainting, such negative experiences were not significantly correlated with the individual level of heat protection measures (*r* = −0.05, *p* = 0.492).

**FIGURE 1:**
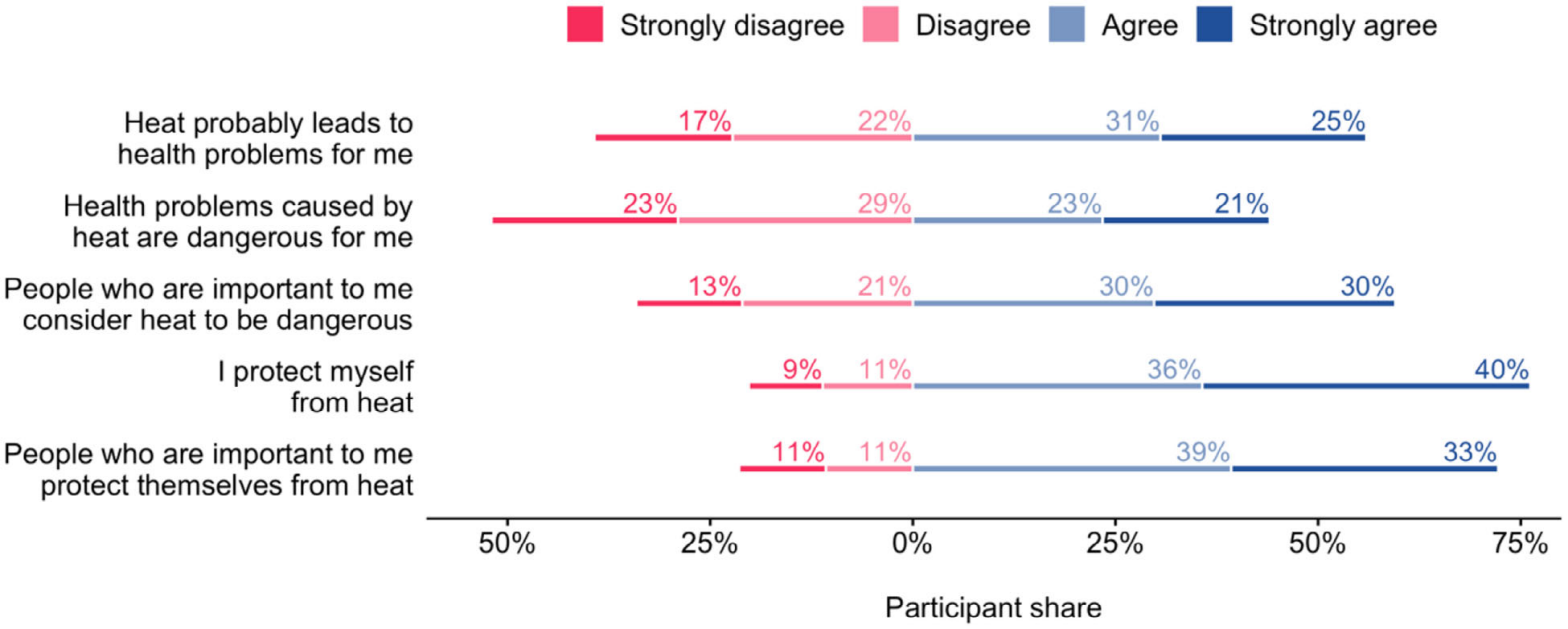
Heat risk perception and protection. Items were not answered by 4%–6% of participants.

#### 3.2.2 Medication

In total, 89 participants (35%) reported taking prescription medications regularly, particularly to treat cardiovascular disease, thyroid problems, diabetes, epilepsy, mental illness, and withdrawal symptoms. A total of 27 participants (11%) were prescribed at least one treatment known to increase heat risks. However, many individuals described their drugs in general terms (e.g., medicine for high blood pressure), due to which effects on heat risks could not be concluded, and the number may be much higher. Among the 89 participants who regularly took medication, only 13 (15%) changed their dosage on hot days, 42 (47%) had the option to store their drugs in a cool place if they wanted to, and 43 reported having their medication with them always, either because they had to take them regularly (*n* = 32, 36%) and/or to prevent theft or loss (*n* = 16, 18%).

#### 3.2.3 Use of and Eviction From Cool Places

As shown in Figure 2, most participants reported using shady spots in public parks, along streets, and pedestrian zones to cool down. Churches, public buildings, such as libraries and government offices, and swimming pools were the least frequented cooling spots. Although 42% were using train and metro stations to cool down, 31% reported being evicted from these locations. At least 20% had also been evicted from stores, shopping centers, streets, pedestrian zones, or public transport services. Eviction was less often observed for churches, swimming pools, and bathing spots by rivers and lakes. The sample included 915 different places to cool down during hot days, and 256 eviction experiences (28%) were reported for these places.

**FIGURE 2:**
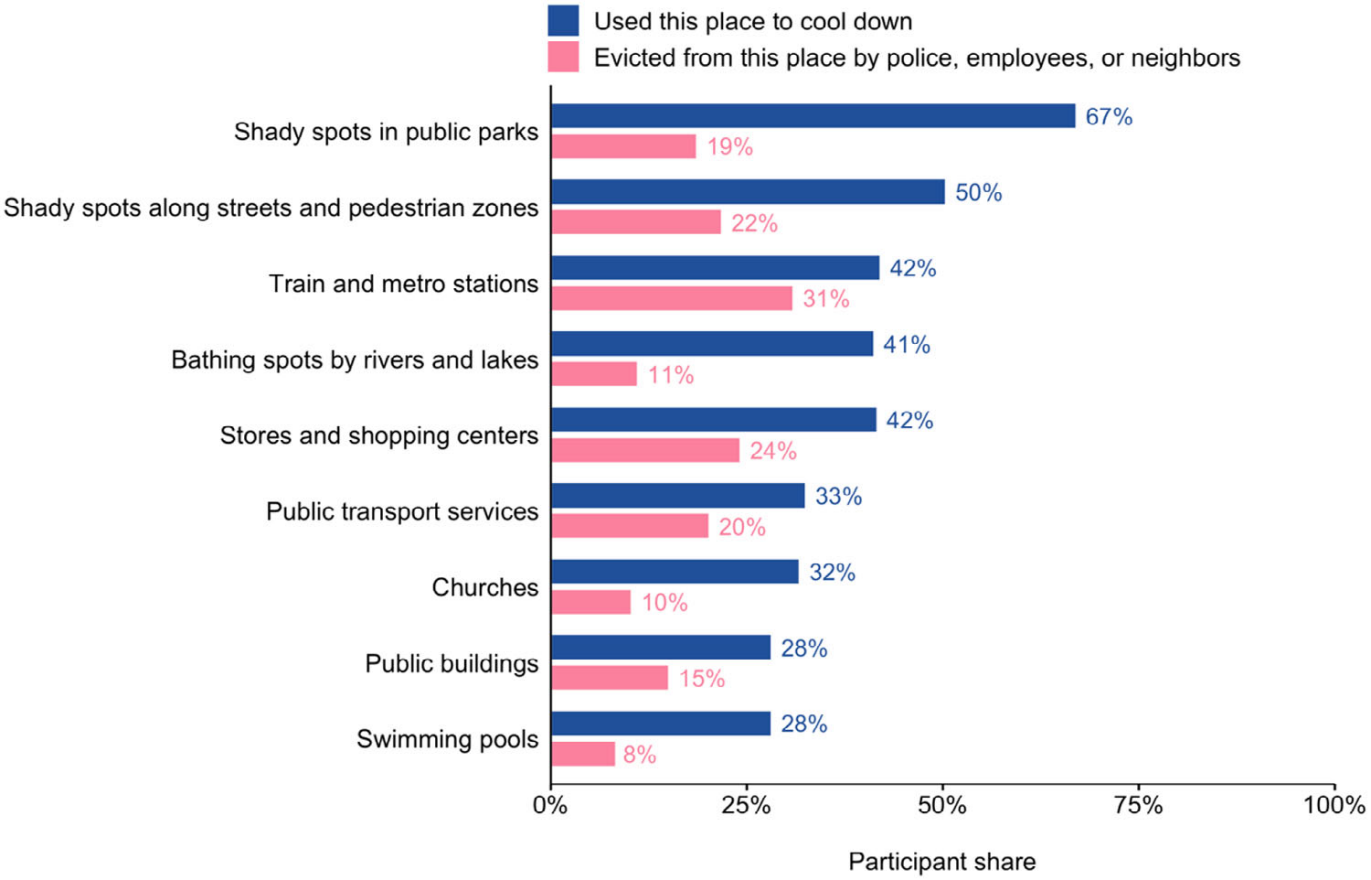
Use of and eviction from cool places. Share of participants stating that they used the listed places or were evicted from there. Answers were missing from 15% to 28% of participants.

#### 3.2.4 Hydration

Although 29% reported drinking 1.5 L or fewer fluids on the previous day, the average consumption of alcohol‐free beverages was relatively high (*M* = 3.09 L, SD = 2.70 L), indicating problems with the assessment of self‐reported drinking quantities. Overall, 81 participants (32%) thought they were not drinking enough. When these participants were asked about the reasons, 51 (63%) reported a lack of money as the primary barrier, 37 (46%) perceived inadequate free supplies as the major reason (such as drinking fountains), and 13 (16%) indicated that they did not like the taste of free supplies. Importantly, 25 participants (31%) deliberately restricted their consumption of fluids to avoid using the toilet frequently. This reason was reported significantly more often by females (75%) compared to males (21%; *χ* ^2^(1, *N* = 79) = 15.01, *p* < 0.001). Participants who reported to protect themselves from heat were not drinking more (*r* = 0.07, *p* = 0.318) but were more likely to perceive their hydration as inadequate (*r* = −0.18, *p* = 0.005).

#### 3.2.5 Clothing and Sun Protection

A majority of participants indicated having enough clothes (60%), access to spaces for storing clothes they did not need (53%), and resources to wash clothes (84%) in the summer. Although most participants (87%) were wearing lightweight, airy clothes at least sometimes, large groups never wore a hat (39%) or long sleeves (39%) to protect themselves from the sun and heat. One‐third of participants (35%) had at least one sunburn since the beginning of the year. Importantly, 55% never used sunscreen, whereas 28% used it sometimes, and 15% used it often. To investigate predictors of sunscreen use, an ordinal logistic regression analysis (Nagelkerke's *R* ^2^ = 0.38) was performed on perceptions of sunburn risk (*Sunburn is dangerous for me*, *M* = 2.66, SD = 1.16), sunscreen efficacy (*Sunscreen helps me to protect myself from sunburn*, *M* = 2.80, SD = 1.10), affordability (*I can afford sunscreen*, *M* = 2.47, SD = 1.20), and pleasantness of use (*I find the use of sunscreen pleasant*, *M* = 2.53, SD = 1.10). Results indicate that participants were more likely to use sunscreen when they perceived it to be effective (OR = 1.54, *p* = 0.021) and pleasant to use (*OR* = 2.02, *p* < 0.001), whereas no significant effects could be found for the other two predictors associated with affordability and perceived sunburn risk (*p* > 0.443; see https://doi.org/10.17605/OSF.IO/RKWAM for details).

#### 3.2.6 Overnight Stay

Many participants spent hot nights in shelter accommodation (35%) or in the open air (33%). Some slept at friends’ or relatives’ homes (21%), in tents (9%), in cars (3%), or in other places (19%). Average sleeping quality was rather bad (*M* = 2.38, SD = 0.95); multiple linear regression (*R* ^2^ = 0.05) revealed that it did not depend on sleeping in specific places except in cars, which was positively related to better‐sleeping quality (*b* = 1.17, *p* = 0.003; see https://doi.org/10.17605/OSF.IO/RKWAM for details).

### 3.3 Discussion

The findings reveal different facets of unhoused people's vulnerability to heat risks. Many have experienced heat‐related fatigue, nausea, or faintness on hot days and take medication that is known to increase heat risks, whereas dosage is rarely adapted. Cool places available in most cities, such as churches or public buildings, are rarely frequented, and considerable shares do not consume enough liquids. Sunburn is common as many forego protective clothing and the use of sunscreen. The individuals’ vulnerability relates to both psychological and systemic factors. Our survey shows that risks of high temperatures are often underestimated, especially in comparison to other people. This may be explained by optimism bias (i.e., the general tendency to believe that negative events are less likely for oneself than others; [34]) but could also stem from comparing potential heat risks with the existing severe conditions many participants reported (e.g., cardiovascular disease, diabetes, mental illness, or low temperatures). Information‐based interventions targeting risk perceptions and appropriate behaviors may help to improve individual heat and sun protection uptake together with adequate structural measures. For instance, our results suggest that communicating the effectiveness of sunscreen could improve the use of free sunscreen offers already available in many German cities. Furthermore, emphasizing how people important to unhoused individuals manage to adapt during heat phases may reduce risky behaviors. Both recommendations are supported by Robertson et al. [27], who found subjective norms and the perceived value of heat information to predict the intention to avoid heat among unhoused respondents in the United States. That difficulty comprehending heat information was also predictive in their data reinforces our point that such communication must be easy to understand. Of course, successful interventions require improving knowledge about risks and behavioral endpoints among all stakeholders working in the field of homelessness, such as social and healthcare workers.

Effective individual adaptation to severe heat and sun requires municipal improvements and policy changes. The survey shows that unhoused people are often evicted from cool places, such as train and metro stations, pedestrian zones, and other shaded public areas. That eviction was reported for 28% of the 915 cooling places named by our participants closely mirrors the mechanism described by Cronley et al. [28], for whom social exclusion in the built environment erodes adaptive capacity by limiting and policing scarce resources and restricting mobility in the United States. One divergence is instructive: Although their participants found public transit to be a source of relief, 31% of our respondents had been evicted from train and metro stations, the very locations often used for cooling. A resource that functions as a refuge in one setting can therefore be effectively withdrawn in another. Municipal heat–health action plans could be revised to prevent law enforcement, shop owners, and neighbors from expelling unhoused individuals during extremely hot days. Furthermore, explicit heat shelters must be created to meet the needs of unhoused populations. Likewise, access to cool, secure storage for medication should be provided, for example, in collaboration with pharmacies. More drinking fountains need to be installed in areas where unhoused people reside, and free drinks should be distributed more often, especially on weekends when many charities and stores are closed. Our findings also underscore the need for more free toilets, especially for women, to increase the acceptance of free drinks for adequate hydration. Clothing banks may further enhance sun protection by providing unhoused people with hats and lightweight long‐sleeved clothing. That 9% of participants spent hot nights in tents is concerning in light of Thompson et al. [29], who measured trapped heat leaving tents up to 5°C warmer than the ambient air at night, exacerbating summer heat exposure. The survey also indicates that emergency shelter accommodation needs to be improved, for instance, by equipping these shelters with air‐conditioning to provide relief from extreme temperatures. However, as indicated in our findings, staying in cars was associated with better sleep, suggesting that shelters must also improve the privacy and safety of unhoused individuals, a long‐standing demand for unhoused individuals’ overall wellness [35].

Taken together, our findings corroborate the emerging evidence on heat and unhoused populations. The central mechanism identified in US samples—that protective action is constrained by the policing and revocability of scarce cooling resources rather than by a lack of awareness [28, 29]—is reproduced here in a European welfare state with universal health coverage (about two‐thirds of the Study 2 sample had health insurance) and a comparatively dense shelter system, indicating that it is not an artifact of any single service landscape. Beyond this, the present studies quantify barriers that have received little attention in previous research: Medication that is neither dosed nor stored accurately, and the deliberate restriction of fluid intake to avoid frequent toilet use. Although results and recommendations are drawn from a large and diverse sample, the findings need to be generalized with caution. Participants were recruited from German day centers, and the experiences of unhoused people who do not engage with support services may differ. The research findings may not be transferable to other regions that feature different climate patterns, social support systems, or homelessness management policies. The apparently high reported liquid intake levels indicate that some items may have been too difficult to answer despite researchers’ attempts to assist the participants in the comprehension and completion of the questionnaire. German‐only questionnaires and substantial missing data (especially for cool place use and eviction) should be considered limitations as well. Future research on unhoused populations should conduct pretesting of survey instruments to ensure data quality. Relatedly, the measures used in this study have not undergone formal psychometric validation; because most constructs were assessed with single items, internal consistency could not be estimated. That the observed associations followed theoretical expectations offers some reassurance, but developing and validating a heat vulnerability instrument for the unhoused remains an important task. Additionally, studies should investigate specific intervention strategies along with structural improvements that result in effective risk reduction for unhoused people during extreme heat waves.

## 4 Conclusion

This research extends evidence on heat vulnerability among unhoused people to a European context through the lens of structural access and social‐cognitive factors. Data indicate that protection against heat was constrained less by the absence of cooling infrastructure than by limited access to it, as unhoused people were evicted from cool places, reported limited access to both hydration access and toilets, and often had no cool or secure place to store medication. At the individual level, self‐reported protection was associated with perceiving that people important to them protect themselves and, to a lesser extent, with perceived severity, whereas previously experienced heat‐related symptoms were not associated with protection. Municipal heat–health action plans should secure access to existing cool places, hydration, and toilets during official heat warnings and deliver communication on protective strategies through peers and service workers. Importantly, these interventions should be designed inclusively, considering unhoused individuals’ needs and circumstances to facilitate engagement and accessibility. Unhoused people's safety from heat‐related health consequences demands public health officials, municipal governments, social service organizations, and community groups to work together to develop sustainable protective strategies. Collaborative approaches integrating insights from affected individuals can structurally enhance resilience in the population's safety during extreme environmental conditions. Given the cross‐sectional and exploratory design, future research should evaluate interventions and develop validated instruments to assess heat vulnerability in this population.

## Author Contributions

**Philipp Sprengholz**: conceptualization, methodology, investigation, formal analysis, visualization, writing – original draft, writing – review and editing, supervision, funding acquisition. **Robert W. Bruckmann**: conceptualization, investigation, writing – review and editing, methodology. **Linnea Noomi Daniel**: data curation, methodology, writing – review and editing. **Lilliana Babette Moreth**: data curation, methodology, writing – review and editing. **Nicole Piatti**: conceptualization, data curation, methodology, writing – review and editing, investigation.

## Funding

This work was supported by the German Federal Ministry of Health (Project HEATCOM, funding number 2523FSB10B).

## Ethics Statement

The study followed the guidelines of the German Psychological Association. Ethical clearance for the study was obtained from the University of Bamberg's IRB (#2024‐04/21).

## Consent

All participants provided informed consent prior to data collection.

## Conflicts of Interest

The authors declare no conflicts of interest.

## Data Availability

The data that support the findings of this study are openly available in OSF at https://doi.org/10.17605/OSF.IO/RKWAM.
